# Early Infant Exposure to Excess Multivitamin: A Risk Factor for Autism?

**DOI:** 10.1155/2013/963697

**Published:** 2013-03-04

**Authors:** Shi-Sheng Zhou, Yi-Ming Zhou, Da Li, Qiang Ma

**Affiliations:** ^1^Institute of Basic Medical Sciences, Medical College, Dalian University, Dalian 116622, China; ^2^Section of Cell Signaling, Okazaki Institute for Integrative Bioscience, National Institutes of Natural Sciences, Okazaki 444-8787, Japan; ^3^Department of Physiology, Institute of Basic Medical Sciences, China Medical University, Shenyang 110001, China; ^4^Department of Neurology, Affiliated Zhongshan Hospital of Dalian University, Dalian 116001, China

## Abstract

Autism, a neurodevelopmental disorder that affects boys more than girls, is often associated with altered levels of monoamines (serotonin and catecholamines), especially elevated serotonin levels. The monoamines act as both neurotransmitters and signaling molecules in the gastrointestinal and immune systems. The evidence related to monoamine metabolism may be summarized as follows: (i) monoamine neurotransmitters are enzymatically degraded/inactivated by three mechanisms: oxidative deamination, methylation, and sulfation. The latter two are limited by the supply of methyl groups and sulfate, respectively. (ii) A decrease in methylation- and sulfation-mediated monoamine inactivation can be compensated by an increase in the oxidative deamination catalyzed by monoamine oxidase, an X-linked enzyme exhibiting higher activity in females than in males. (iii) Vitamins can, on one hand, facilitate the synthesis of monoamine neurotransmitters and, on the other hand, inhibit their inactivation by competing for methylation and sulfation. Therefore, we postulate that excess multivitamin feeding in early infancy, which has become very popular over the past few decades, may be a potential risk factor for disturbed monoamine metabolism. In this paper, we will focus on the relationship between excess multivitamin exposure and the inactivation/degradation of monoamine neurotransmitters and its possible role in the development of autism.

## 1. Introduction

Autism is a neurodevelopmental disorder that appears in the first three years of life, affecting boys more than girls in a ratio of approximately 4 : 1 [1]. One of the most consistent abnormalities in autism in the published literature since 1961 is elevated blood serotonin (see [2] for review). Autism may also be associated with altered metabolism of catecholamines (dopamine, norepinephrine, and epinephrine), for example, elevated plasma levels of dopamine and epinephrine [3]. The monoamines (serotonin and catecholamines) are known to act not only as neurotransmitters, but also as signaling molecules in the gastrointestinal tract and immune system. Moreover, neurotransmitters may play a role in neurogenesis during brain development [4]. Thus, abnormal monoamine metabolism may have a profound impact on immune responses and gastrointestinal activities [5–7] as well as on neurodevelopment [8, 9]. From this point of view, it seems that disturbed monoamine metabolism, which is known to be caused by a variety of factors (e.g., drugs [9] and diet [10]), may play a crucial role in the development of autism. Thus, a better understanding of the mechanism of disturbed monoamine metabolism may provide insights into the etiology of autism.

Evidence suggests that the etiology of autism may involve both genetic and environmental factors [11, 12]. However, exactly what those environmental factors are remains to be determined. Notably, there were no significant pollution events in the United States from the 1980s and through the 1990s, but why was there a sudden increase in the incidence of autism among the 1987–1992 birth cohorts [13, 14]? If disturbed monoamine-neurotransmitter metabolism plays a role in the development of autism, factors accounting for the increased prevalence of autism could be those that can directly or indirectly affect monoamine-neurotransmitter metabolism. Some vitamins are known to increase the levels of monoamine neurotransmitters (see below). Excess vitamins are also known to have side effects like neurotoxicity [15]. Most significantly, over the past few decades, there has been a significant increase in multivitamin exposure in infancy due to high vitamin feeding and supplementation [16, 17]. Thus, the possibility exists that the increased incidence of autism may be related to excess multivitamin exposure. In this paper, we will focus on the relationship between excess multivitamin exposure and the inactivation/degradation of monoamine neurotransmitters and its possible role in the development of autism.

## 2. Monoamine-Neurotransmitter Inactivation

**Figure 1:**
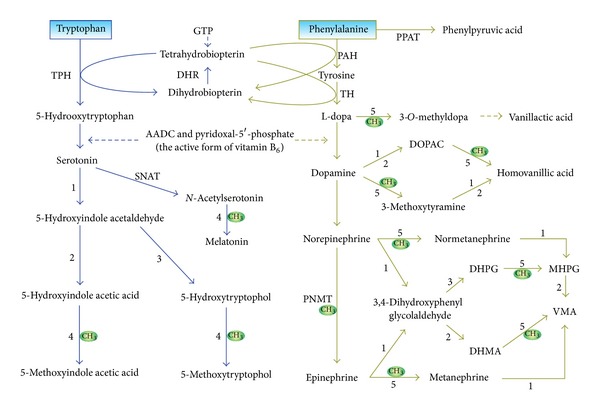
The synthesis and degradation of serotonin and catecholamines. Oxidative deamination and methylation are two key mechanisms for inactivating monoamine neurotransmitters. Note that without labile methyl groups, methylation-mediated degradation of serotonin and catecholamines cannot take place, even though the methyltransferases involved are normal. 1, monoamine oxidase; 2, aldehyde dehydrogenase; 3, aldehyde reductase; 4, acetylserotonin *O*-methyltransferase; 5, catechol-*O*-methyltransferase. AADC: aromatic L-amino acid decarboxylase; DHMA: 3,4-dihydroxymandelic acid; DHPG: 3,4-dihydroxyphenylglycol; DHR: dihydropteridine reductase; DOPAC: 3,4-dihydroxyphenylacetic acid; GTP: guanosine triphosphate; MHPG: 3-methoxy-4-hydroxyphenylglycol; PAH: phenylalanine hydroxylase; PNMT: phenylethanolamine *N*-methyltransferase; PPAT: phenylalanine(histidine):pyruvate aminotransferase; SNAT: serotonin *N*-acetyltransferase; TH, tyrosine-3-hydroxylase; TPH, tryptophan-5-hydroxylase; VMA: vanillylmandelic acid.

It is known that to ensure normal functioning of the nervous, immune, and digestive systems, the monoamines released from the nervous system and the gastrointestinal tract must be inactivated/degraded and eliminated in time. Monoamine-neurotransmitters, like xenobiotics (substances foreign to the body, such as pollutants, food additives, pesticides, and drugs), are metabolized through enzymatic phase I (oxidation, reduction, and hydrolysis) and phase II reactions (conjugation, e.g., methylation, sulfation, acetylation, glucuronidation, and glutathione conjugation) [18]. The characteristics of monoamine degradation are as follows: (1) enzymatic degradation: the degradation of all the monoamines and their precursor amino acids is enzymatic multipathway and multistep processes (Figure 1). The major enzymes involved in the degradation of monoamine neurotransmitters are monoamine oxidase (MAO), catechol-*O*-methyltransferase (COMT), acetylserotonin *O*-methyltransferase, and sulfotransferases, which are responsible for the oxidative deamination, methylation, and sulfation of the neurotransmitters, respectively. Genetic polymorphism of the enzymes has been demonstrated to contribute to interindividual differences in the overall metabolism of monoamines [19, 20]. In the degradation of monoamines and their precursors, when one pathway is interrupted, another pathway can partially compensate. For example, when the phenylalanine-tyrosine pathway is blocked by phenylalanine hydroxylase deficiency, phenylalanine is converted to phenylpyruvate, resulting in phenylketonuria [21]. 

**Figure 2:**
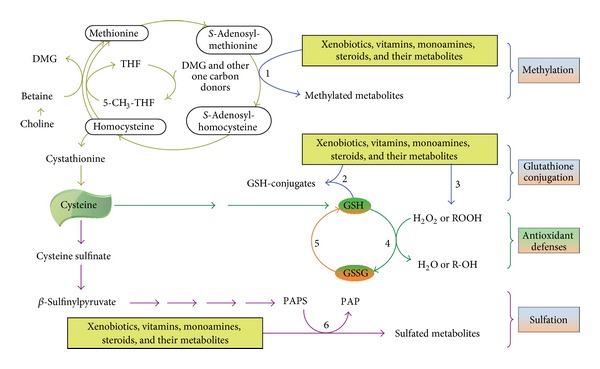
Role of methyl groups and sulfur amino acids in the detoxification system. Methylation, sulfation, glutathione conjugation, and glutathione-mediated antioxidant defense are major components of the detoxification system. An adequate supply of methyl donors, primarily betaine and choline, is a prerequisite for methylation reactions. Glutathione, formed from cysteine, is required for glutathione conjugation and the clearance of hydrogen peroxide and hydroxyl radicals, while 3′-phosphoadenosine 5′-phosphosulfate (PAPS), derived from the metabolism of sulfur amino acids, is the universal sulfate donor for all sulfation processes. Excess vitamins may disturb the degradation of monoamine neurotransmitters by depleting the body's methyl-group and sulfate pools. 1, methyltransferases; 2, glutathione *S*-transferase; 3, phase I xenobiotic-metabolizing enzymes; 4, glutathione peroxidases; 5, glutathione reductase; 6, sulfotransferases. 5-CH_3_-THF: 5-methyltetrahydrofolate; DMG: dimethylglycine; GSH: reduced glutathione; GSSG: oxidized glutathione; PAP: 3′-phosphoadenosine-5′-phosphate; R-OH: alcohols; ROOH: organic peroxides (which generate free radicals); THF: tetrahydrofolate.

(2) Need for methyl groups and sulfate: as shown in Figure 2, methyl groups and sulfur amino acids (e.g., methionine and cysteine) are required for the body's detoxification and antioxidant activities (Figure 2). An adequate supply of methyl groups and sulfate is prerequisite for methylation- and sulfation-mediated monoamine-neurotransmitter inactivation. Since both the biotransformation of exogenous chemicals and the degradation of monoamine neurotransmitters share the same pool of methyl groups [22] and sulfate [23], in theory, any chemicals (such as vitamins, see the following) that consume methyl groups and/or sulfur amino acids in their biotransformation may competitively inhibit the methylation and sulfation of monoamine neurotransmitters. 

(3) Gender differences in monoamine-neurotransmitter inactivation: as mentioned above, monoamine neurotransmitters can be inactivated either by deamination, by methylation, or by sulfation. The redundant nature of monoamine-neurotransmitter metabolism enables one pathway to compensate for blockade of the other. For example, reduced or absent activity of MAO leads to a decrease in the production of deaminated metabolites and an increase in that of *O*-methylated amine metabolites [24, 25], while inhibition of COMT increases the production of 3,4-dihydroxyphenylacetic acid [25], a deaminated metabolite of dopamine (Figure 1). Thus, if methylation and sulfation cannot take place (e.g., due to depleted methyl-group and sulfate pools by exogenous chemicals) [22, 23], the inactivation of monoamine neurotransmitters will depend mainly on the activity of MAO. Importantly, the genes encoding the two isoforms of MAO are X-linked [26], and their activity is lower in males than in females [27, 28], suggesting a biological basis of sex differences in monoamine degradation. Such a sex difference in MAO activity also suggests that males might have less ability to compensate for blockade of methylation- and sulfation-mediated monoamine inactivation than females. Therefore, it is conceivable that similar levels of exogenous chemical exposure may disturb the inactivation of monoamine neurotransmitters in males more than in females.

## 3. Effect of Vitamins on Monoamine-Neurotransmitter Metabolism

Excess vitamins, like xenobiotics and monoamine neurotransmitters, are also degraded through phase I and phase II reactions and thus may increase the consumption of labile methyl-groups and sulfate. Moreover, some vitamins are known to play a role in the synthesis of monoamine neurotransmitters. For example, vitamin B_6_ is a cofactor for aromatic L-amino acid decarboxylase that catalyzes the formation of serotonin and dopamine (Figure 1), while 5-methyltetrahydrofolate, the active form of folate, also stimulates the synthesis of monoamine neurotransmitters [29]. Therefore, excess vitamins can increase the levels of monoamine neurotransmitters either by competing for the same biotransformation system or by facilitating the synthesis, or by both. Indeed, evidence shows that high doses of vitamin C decrease plasma-conjugated dopamine and norepinephrine levels by competing for sulfation [30], whereas nicotinamide increases the levels of plasma of norepinephrine [31], serotonin, and histamine [32], presumably due to a decrease in methylation-mediated degradation of the monoamines. Vitamin B_6_ supplementation can increase the blood serotonin levels of newborn babies [33]. Interestingly, Berman and colleagues [34] found that maternal supplementation with vitamin B_6_ during the last 3 to 5 weeks of pregnancy increased the maternal blood levels of serotonin at parturition but did not increase the cord blood serotonin level or urinary 5-hydroxyindoleacetic acid output in the newborn infants, suggesting that the placenta may protect the fetus from the risk of excess vitamin exposure. Although little is known about the effect of excess vitamin exposure on the neurotransmitter metabolism in the human infant brain, evidence from animal studies has shown that some vitamins can affect the metabolism of central monoamine neurotransmitters. For example, vitamin C [35] and vitamin B_6_ [36, 37] increase the levels of serotonin in the brain of rats. Recently, Tekes and colleagues [38] found that neonatal vitamin A or vitamin D treatment has significant influence on the metabolism of monoamine neurotransmitters in the adult rat brain. Therefore, excess vitamin exposure may be a potential risk factor for neurotransmitter metabolism disorders.

## 4. Toxicity of Excess Vitamins

It has been known for over a century that the dose-response curve for many micronutrients is nonmonotonic, having an initial stage of increasing benefits with increased intake, followed by increasing costs as excesses become toxic [39]. Both vitamin deficiency and vitamin excess are known to cause toxicity, including neurotoxicity [15, 40]. A meta-analysis of randomized trials of antioxidant supplements for primary and secondary prevention suggests that supplementation of vitamin A and E may increase mortality [41]. Supplemental folic acid (the synthetic form of folate) was also found to be associated with increased mortality [42, 43]. Davis and colleagues [44] found an association between high serum thiamine levels and sudden infant death syndrome (SIDS, a sudden and unexplained infant death most likely to occur between 2 and 4 months of age), and they further demonstrated that high doses of thiamine could cause death in rabbits and mice due to respiratory failure. Moreover, there is evidence suggesting an association between early infant vitamin supplementation and an increased risk of allergic diseases later in life [45, 46]. Although these data are not conclusive, they at least suggest the possibility that excess vitamin exposure may lead to serious health outcomes.

To date, little is known about the relationship between early infant exposure to excess vitamins and autism, except a recent hypothesis that suggests that excess folic acid supplementation may be a risk factor for autism [47]. There are two studies that examine the relationship between early vitamin exposure and learning development in rats. One found that neonatal vitamin A exposure may induce a long-lasting defect in learning [48], and the other showed that niacin supplementation induced spatial learning impairment in rats [49]. These observations suggest that early excess vitamin exposure may have adverse effects on neurodevelopment. It should be noted that the neurological effects of vitamin deficiency and vitamin excess may be similar [15]. Such a similarity could be a common cause for a wrong diagnosis. For example, SIDS was initially suggested to be related to a thiamine deficiency. To test this hypothesis, Davis and colleagues [44] compared serum thiamine levels between 233 SIDS infants and 46 control infants dying from other causes. Unexpectedly, they found that most of the SIDS infants had significantly higher serum thiamine levels. Therefore, to avoid making a wrong diagnosis, the levels of vitamins and their metabolites should be monitored.

It should be pointed out that some cofactors, although not belonging to vitamins, may also play an important role in the synthesis of monoamine neurotransmitters. As shown in Figure 1, tetrahydrobiopterin, which is synthesized from guanosine triphosphate, is an essential cofactor for dopamine and serotonin biosynthesis. Thus, it is conceivable that excess tetrahydrobiopterin can increase monoamine-neurotransmitter levels and may contribute to monoamine-related mental disorders. Indeed, evidence has shown that tetrahydrobiopterin may cause preferential death of catecholaminergic cells, presumably due to increased dopamine levels [50]. However, it is unlikely that tetrahydrobiopterin may play a role in the rapidly increased prevalence of autism in the past few decades, since there is no evidence suggesting an increase in the synthesis of tetrahydrobiopterin in the early infancy of autistic patients, or in the content of tetrahydrobiopterin in infant foods.

## 5. High Multivitamin Exposure and Increased Autism Prevalence

**Figure 3:**
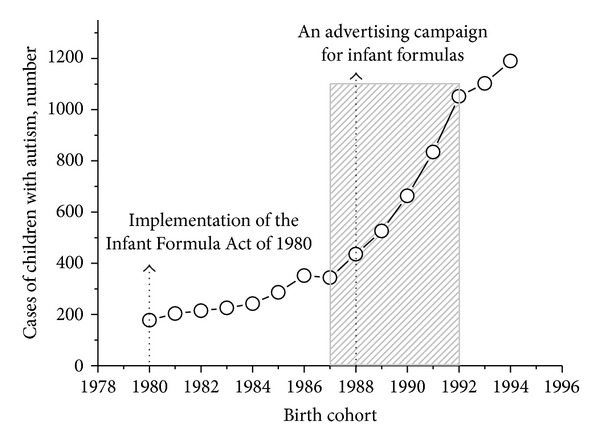
Events related to infant feeding and the prevalence of autism. The data on the number of cases of children with autism in the 1980–1994 birth cohorts in California are taken from [13]. Note that the implementation the Infant Formula Act of 1980 [51] was followed by an apparent increase in autism incidence, while the advertising of infant formula to the general public, started in 1988, was accompanied with a rapid increase in the incidence of autism. The increases were greatest for annual cohorts born from 1987 to 1992.

For decades, since it was first described by Kanner in 1943 [52], the prevalence of autism in the United States was low. Autism prevalence studies published before 1985 showed prevalence rates of 4 to 5 per 10,000 children for the broader autism spectrum and about 2 per 10,000 for the classic autism definition [53]. Since 1985, there have been higher rates of autism, with the greatest annual increases occurring between the 1987 and 1992 birth cohorts [14]. During this period, there were no significant national environmental events but a significant event related to infant feeding. In 1988, US formula companies removed the realm of infant feeding from the exclusive supervision of the medical profession and targeted an advertising campaign for their formula products at the general public [51]. If formulas played a role in increased autism prevalence, the campaign should have been followed by several consecutive years of rapid increase in autism cases. In fact, the sharp increase in autism prevalence in annual cohorts born from 1987 to 1992 in California (Figure 3) and the whole of the United States [14] occurred simultaneously with the initiation of the direct advertising of infant formula. The prevalence of autism among 6-year-old children increased from 4.6 per 10,000 in the 1986 birth cohort to 19.1 per 10,000 in the 1992 birth cohort in the United States [14]. Moreover, there are studies showing that premature weaning and suboptimal breast-feeding practices are associated with increased risk of autism [54, 55]. Thus, it appears that the risk factor(s) for autism may be present in infant foods.

Among the possible risk factors in infant foods, such as nutritional imbalances (deficiencies and excesses) and food additives and contaminates, excess multivitamin exposure may be the most common and important. In order to insure the safety of formulas, the United States implemented the Infant Formula Act of 1980, which sets a lower limit of vitamins in infant formulas but does not set an upper limit for most vitamins [56]. This has caused concern that, without upper limits, super-fortified formula could be produced [57] and may have a direct toxic effect [58]. In fact, infant formulas, especially those for premature infants, generally contain much higher levels of vitamins. For example, the content of niacin, folic acid, vitamin B_6_, thiamine, and vitamin C in a premature infant formula (see, http://abbottnutrition.com/products/similac-special-care-20-with-iron) is about 20 (5,000 versus 250 *μ*g/100 kcal), 9 (37 versus 4 *μ*g/100 kcal), 7 (250 versus 35 *μ*g/100 kcal), 6 (250 versus 40 *μ*g/100 kcal), and 4 (37 versus 8 mg/100 kcal) times the lower limit value, respectively. The level of thiamine in some manufactured milk-based formulae (2160 *μ*g/L) was found to be about 20 times that of human breast milk (mean 178 *μ*g/L) [44]. In addition to the vitamin supplementation of infant formula, multivitamin use in infants and toddlers is very common [16]. Thus, high-vitamin feeding may increase the risk of vitamin overload. Indeed, many studies have shown that formula-fed infants have higher levels plasma/serum of vitamins than human milk-fed infants [59–62]. Unmetabolized folic acid, a sign of folic acid overload, is observed in the serum of 4-day-old infants fed with formula [63]. Porcelli and colleagues [62] found a several times increase in the plasma levels of riboflavin and pyridoxine and a more than 10 times increase in the urine riboflavin and pyridoxine concentrations in very low-birth-weight neonates after being fed with preterm infant formula. Baeckert and colleagues [64] showed that very low-birth-weight infants who received the recommended parenteral vitamin supplement as part of their total parenteral nutrition developed elevated plasma riboflavin concentrations during their first postnatal month with peak concentrations 100-fold above baseline umbilical cord plasma vitamin concentrations. Moreover, there are two studies finding high plasma levels and urinary excretion of methylated metabolites of niacin in autistic patients [65, 66], which suggests a niacin overload, because excess niacin is rapidly degraded after ingestion, but its methylated metabolites remain longer in the circulation [31, 67]. Given that excess vitamins may lead to neurotoxicity and disturbances in monoamine neurotransmitter metabolism, as mentioned earlier, it is possible that high multivitamin exposure may play a role in the increased prevalence of autism. 

## 6. Critical Window of Vulnerability for Autism

Brain overgrowth has been noted among children with autism [68]. An understanding of the timing of brain enlargement in autism may be particularly helpful in identifying the window of vulnerability for autism. Hazlett and colleagues [69] observed generalized cerebral cortical enlargement in individuals with autism at both 2 and 4 to 5 years of age, but they found that there was no significant difference from controls in the rate of brain growth for this age interval, indicating that brain enlargement in autism results from an increased rate of brain growth before age of 2 years. A recent study using diffusion tensor imaging showed that there have been significant brain differences at age of 6 months between high-risk infants who later develop autism and those who did not [70], clearly indicating that autism develops over time during infancy. Moreover, studies found that premature weaning and suboptimal breast-feeding practices may increase the risk of autism [54, 55]. The above two lines of evidence suggest that the first few months after birth could be a critical window of vulnerability for autism.

Current understanding of the rates of maturation of metabolic capability indicates that human infants up to approximately 6 months of age are typically more sensitive to chemical toxicity than adults due to their immature detoxification systems [71]. This suggests that newborn infants, especially premature infants, may have a low tolerance to excess vitamins. Indeed, available evidence, although limited, has shown an association between high levels of some vitamins (thiamine [44] and vitamin C [72]) and apparent life-threatening events and SIDS in infancy. A randomized controlled trial on vitamin C supplementation in very preterm infants also showed that infants who died during the trial were those who had significantly higher vitamin C concentrations before randomization than surviving infants [73]. Evidence from animal studies suggests that high exposure to vitamin A [48] and niacin [49] in the early life has adverse effects on the behaviors of adult rats. Thus, it appears that high multivitamin feeding in the first few months of life may be particularly harmful. Although there is little information on the role of excess vitamins in infant brain injuries, it is common knowledge that chemical exposure-induced neurological injury may have a variety of manifestations, depending on the length and degree of exposure [74]. Notably, preterm birth is associated with increased risk for both autism and other neurological conditions, such as cognitive, visual, and hearing impairments; and there is considerable cooccurrence of autism with other neurological and cognitive disorders [75]. We therefore postulate that autism might be one of chemical/excess-vitamin exposure-induced neurological sequelae (which may range from neurological deficits to death) in early infancy. What is worthy of note is that with the maturation of metabolic function and age-related changes in feeding foods, the causal exposure present in infancy and resultant metabolic and neurological manifestations may no longer exist. This may account for why there is lack of consistent biological markers in autism. Even elevated blood serotonin, the most consistent serotonin-related finding in autism, may not be observed in adolescent autistic patients [76].

## 7. Conclusion

Given that (1) high multivitamin feeding is very common in early infancy, (2) excess vitamins may cause neurotoxicity and disturb monoamine-neurotransmitter metabolism, and (3) autism is often associated with abnormal levels of monoamine neurotransmitters, it seems that excess multivitamin exposure in early infancy may be a potential risk factor for autism. Further studies are needed to confirm this hypothesis.
